# Traumatic Tetanus Treated with Eserine

**Published:** 1882-10

**Authors:** 


### TRAUMATIC TETANUS TREATED WITH ESERINE.

Dr. Thomas Layton reports a case of tetanus occurring in a boy, aged eleven years, following, after an interval of three weeks, the wounding of the sole of the foot with a splinter. Chloral, bromide of potassium, and cannabis indica were employed without benefit. Eserine was then administered in doses of 1-64th grain every hour. Recovery took place. Dr. Layton calls attention to the following points :

1. The child took a full adult dose of sulphate of eserine every hour for several days, and not only were there at no time symptoms of poisoning, but the beneficial action of the remedy was apparently manifest.

2. There was never the least contraction of the pupils. On two occasions, as mentioned in the observation, the pupils were dilated ; at all other times they responded to light in a normal manner.

3. It was noticed that the sulphate of eserine increased either the secretion of tears and saliva, or defectation ; with regard to the last, an occasional purgative had to be employed during the progress of the case.—*New Orleans Med. and Surg. Jour.*

